# Diosmin Mitigates Gentamicin-Induced Nephrotoxicity in Rats: Insights on miR-21 and -155 Expression, Nrf2/HO-1 and p38-MAPK/NF-κB Pathways

**DOI:** 10.3390/toxics11010048

**Published:** 2023-01-01

**Authors:** Rania I. Nadeem, Amany S. Aboutaleb, Nancy S. Younis, Hebatalla I. Ahmed

**Affiliations:** 1 Pharmacology and Toxicology Department, Faculty of Pharmacy, Heliopolis University, Cairo 11785, Egypt; 2 Pharmacology and Toxicology Department, Faculty of Pharmacy (Girls), Al-Azhar University, Cairo 11754, Egypt; 3 Pharmaceutical Sciences Department, Faculty of Clinical Pharmacy, King Faisal University, Al-Ahsa, Al-Hofuf 31982, Saudi Arabia

**Keywords:** diosmin, gentamicin, nephrotoxicity, miR-21, miR-155, p38-MAPK, NF-κB

## Abstract

Gentamicin (GNT) is the most frequently used aminoglycoside. However, its therapeutic efficacy is limited due to nephrotoxicity. Thus, the potential anticipatory effect of Diosmin (DIOS) against GNT-prompted kidney damage in rats together with the putative nephroprotective pathways were scrutinized. Four groups of rats were used: (1) control; (2) GNT only; (3) GNT plus DIOS; and (4) DIOS only. Nephrotoxicity was elucidated, and the microRNA-21 (miR-21) and microRNA-155 (miR-155) expression and Nrf2/HO-1 and p38-MAPK/NF-κB pathways were assessed. GNT provoked an upsurge in the relative kidney weight and serum level of urea, creatinine, and KIM-1. The MDA level was markedly boosted, with a decline in the level of TAC, SOD, HO-1, and Nrf2 expression in the renal tissue. Additionally, GNT exhibited a notable amplification in TNF-α, IL-1β, NF-κB p65, and p38-MAPK kidney levels. Moreover, caspase-3 and BAX expression were elevated, whereas the Bcl-2 level was reduced. Furthermore, GNT resulted in the down-regulation of miR-21 expression along with an up-regulation of the miR-155 expression. Histological examination revealed inflammation, degradation, and necrosis. GNT-provoked pathological abnormalities were reversed by DIOS treatment, which restored normal kidney architecture. Hence, regulating miR-21 and -155 expression and modulating Nrf2/HO-1 and p38-MAPK/NF-κB pathways could take a vital part in mediating the reno-protective effect of DIOS.

## 1. Introduction

Aminoglycosides are a class of potent, broad-spectrum antibiotics employed to defend against infections triggered by gram-negative microorganisms. Due to its low cost and consistent potency, gentamicin (GNT) is the most often utilized aminoglycoside [1]. However, its therapeutic efficacy is limited due to the induction of nephrotoxicity [2]. Severe proximal renal tubular necrosis as well as elevated serum creatinine and urea levels are hall markers of GNT nephrotoxicity [3]. Additionally, Kidney Injury Molecule-1 (KIM-1), a tubular protein, is documented as a highly delicate and specific biomarker to detect the early stages of GNT nephrotoxicity [4].

The precise mechanism by which GNT induces renal damage remains to be fully elucidated. GNT selectively accumulates in the renal proximal tubular cells, causing glomerular deterioration, cellular necrosis, and fibrosis, as well as inflammation. It has been stated that the engendering of reactive oxygen species (ROS), inflammation and apoptosis take part in the pathogenesis of GNT-induced kidney toxicity [5,6,7].

Remarkably, the antioxidant defense mechanism’s chief regulator, nuclear factor erythroid 2-related factor 2 (Nrf2), maintains the production of antioxidant genes such as heme oxygenase-1 (HO-1) and halts oxidative damage in cells [8]. Targeting the Nrf2/HO-1 pathway is proposed as a potential protective pathway against GNT-prompted nephrotoxicity by a significant body of research [9,10,11]. 

Furthermore, GNT-nephrotoxicity has been linked to renal tissue up-regulation of p38-mitogen-activated protein kinase (p38-MAPK) and nuclear factor kappa B (NFB); both of which are imperative in the inflammatory and apoptotic pathways [12,13,14,15]. 

microRNAs (miRNAs), small non-coding RNA molecules, are engaged in plentiful physiological and pathological processes by regulating gene expression [16,17,18]. They are readily detected in body fluids and are now being explored as clinical diagnostics and therapeutic targets [19,20]. In the kidneys, miRNAs have been associated with normal physiology and development as well as the pathogenesis of several renal diseases, such as renal carcinoma, diabetic nephropathy, acute kidney injury and others [21]. In fact, many studies have demonstrated the association between kidney disease and miRNAs in humans and experimental animals [22,23,24,25,26].

Diosmin (3′,5,7-trihydroxy-4′-methoxyflavone 7-rhamnoglucoside) (DIOS), a citrus fruit unsaturated flavonoid glycoside, is a semi-synthetic drug used to treat venous diseases, such as chronic venous insufficiency and varicose veins [27]. DIOS is acknowledged to possess several pharmacological effects, comprising anti-inflammatory, antioxidant, and anti-apoptotic activities [28,29,30]. Furthermore, DIOS has anti-diabetic [31], anti-cancer [32], liver-protective [29], neuroprotective [33], and reno-protective properties [34,35]. Indeed, DIOS has been proven to defend the kidneys in various models of renal damage via anti-oxidant, anti-inflammatory and anti-apoptotic pathways [28,36,37,38]. Interestingly, a recent study conducted by Ali et al. [39] demonstrated the nephroprotective effect of DIOS against gentamicin-induced nephrotoxicity via the suppression of oxidative stress. However, the anti-inflammatory and antiapoptotic effects of DIOS as well as its impact on miRNAs against GNT nephrotoxicity have not yet been explored. 

Towards recapitulation, our work pursues to divulge the possible protective outcome of DIOS versus GNT-prompted nephrotoxicity in rats and to unveil the putative mechanisms underlying this effect. 

## 2. Materials and Methods

### 2.1. Drugs and Chemicals

Gentamicin (GNT), DIOS, and all utilized chemicals were brought from Sigma-Aldrich, St. Louis, MO, USA. DIOS was suspended in the oral vehicle, 0.5% carboxymethyl cellulose (CMC), while saline was the vehicle for dissolving GNT. All used chemicals were of top quality and analytical grade.

### 2.2. Animals 

Thirty-two adult male Sprague-Dawley rats were procured from the Nile Co. for Pharmaceuticals and Chemical Industries, Cairo, Egypt. At the beginning of the experiment, the rats weighed approximately 150–300 g. Animals were arbitrarily alienated into four groups (four rats/stainless-steel cage) in the animal house of the Faculty of Pharmacy (Girls), Al-Azhar University facilitated with suitable controlled temperature (25 ± 2 °C), and humidity. A 12 h artificial light/dark cycle was applied, and the rats had unrestricted access to water and commercial standard chow pellets. All experiments in this study were accomplished in agreement with the ethical procedures and policies approved by the Ethics Committee of the Faculty of Pharmacy, (Girls), Al-Azhar University (Approval number: 318) and encountered with the terms of the US National Institutes of Health guide for the care and use of laboratory animals (NIH Publications No. 85-23, revised 2011).

### 2.3. Experiment Design 

After acclimatization for a week, rats were erratically allotted into four equal groups, including eight rats; groups and treatments were given as follows: 

Control group: rats received 0.5% CMC (1 mL/animal P.O.) for 14 days and saline (1 mL/animal, I.P.) from the 8th to the 14th day. 

GNT group: rats received 0.5% CMC (1 mL/animal, P.O.) for 14 days and GNT (100 mg/kg, I.P.) was administered from the 8th to the 14th day [15]. 

GNT + DIOS group: rats received DIOS (100 mg/kg P.O) for 14 days [40] and GNT was administered one hour after DIOS oral administration commencing from the 8th to the 14th day.

DIOS group: rats were given DIOS (100 mg/kg, P.O.) for 14 days and a concomitant dose of saline (1 mL/animal I.P.) commencing from the 8th to the 14th day.

The weight of the rats was measured twenty-four hours following the last injection, and blood samples were taken while being anesthetized with pentobarbital (50 mg/kg) [41] through the retro-orbital plexus. Serum was isolated and used for biochemical assessment of kidney function biomarkers. Rats were then euthanized, and the kidneys were promptly removed, weighted, and rinsed in ice-cold normal saline (0.9% *w*/*v*). The kidneys were split into two identical pieces: one half was stored at −80 °C for further biochemical investigation. The other half was fixed in 10% buffered formalin for histopathological investigations. For biochemical investigation, the tissues were homogenized, with an ultimate concentration of 10% *w*/*v*, in ice-cold 0.1 M phosphate buffer (pH 7.4). 

### 2.4. Methods

#### 2.4.1. Estimation of Relative Kidney Weight

The relative kidney weight was estimated following the below equation [42]: 

The kidney weight divided by body weight (g/g) × 100 yields the relative kidney weight.

#### 2.4.2. Measurement of Total Protein

The quantitative protein analysis was done with the Bradford Protein Assay Kit (BIO BASIC INC. Markham, ON, L3R 8T4, Canada). The assay was performed according to the manufacturer’s guidelines.

#### 2.4.3. Biochemical Investigation 

The assessment of renal function in serum and oxidative stress markers in the kidney was done by quantitative colorimetric assay with an ultraviolet (UV)—visible spectrophotometer (Shimadzu UV-1601, 84, Tokyo, Japan), and the commercially available test kits were supplied by (Biodiagnostic, Giza, Egypt), according to the manufacturer’s guidelines. BUN was measured by its action with a chromogenic reagent that forms a colored complex [43], while SCr was assessed using a picrate which forms a red-colored complex with creatinine [44]. Lipid peroxidation was measured as MDA by its reaction with thiobarbituric acid [45]. SOD enzyme activity was estimated by its ability to inhibit the phenazine methosulphate-mediated reduction of nitro blue tetrazolium dye [46]. TAC was assessed according to Koracevic et al. [47].

#### 2.4.4. Enzyme-Linked Immunosorbent Assay (ELISA)

ELISA kits from MyBioSource, San Diego, CA, USA (catalog no. MBS355395) were used to define KIM-1 in the serum and tissue levels of HO-1, p38-MAPK, and IL-1β (catalogs no. MBS764989, MBS765087 and MBS825017 respectively) following the manufacturer’s directions. Furthermore, the tissue levels of TNF-α, BAX, and BCL2 were assessed using Rat ELISA Kit [Cusabio Biotech, Chins; CSB-E11987r, CSB-EL002573RA and CSB-E08854r, respectively] according to manufacturer’s instructions.

#### 2.4.5. Western Blotting

Lysis of the renal tissues’ homogenate was achieved with an ice-cold lysis buffer (10% glycerol, 2% SDS in 62 mM Tris-HCl, pH 6.8), which contains a protease inhibitor cocktail (Sigma-Aldrich, St. Louis, MO, USA). Protein content in the composed protein lysates was done using bovine serum albumin as a standard according to the Bradford method [48]. Under denaturing conditions, an equal amount of total protein (7.5 μg) was undertaken by sodium dodecyl sulfate-polyacrylamide gel electrophoresis (SDS-PAGE), transmitted onto nitrocellulose membranes, and then blocked with 6% non-fat dry milk in TBS-Tween buffer for 3 h at 4 °C. The nitrocellulose membranes were incubated with the specific primary antibody against the detected protein [anti-NF-κB p65 (1:1000; Cell Signaling Technology, Danvers, MA, USA; Cat. No. 3034), anti-Nrf2 (1:500; Abcam (Cambridge, UK); Cat. No. ab137550), anti-Caspase-3 (1:1000; Cell Signaling Technology (USA); Cat. No. 9662)], at 4 °C overnight. A β-actin antibody, the housekeeping gene, was added the following day and incubated at 4 °C for 1 h on a roller shaker. Finally, the membranes were washed five times for 5 min each in Tris-buffered saline-tween 20 (TBST) and incubated with Horseradish Peroxidase (HRP)-conjugated secondary antibody (1:2000; Novus Biologicals, Centennial, CO, USA; Cat. No. NBP1-75297) for 1 h at room temperature. Furthermore, rinsing of the membranes with TBST buffer was done, scanning of the bands with the ChemiDoc scanner was performed, and the densitometric intensity of each band was quantified after normalization with the housekeeping gene expression.

#### 2.4.6. Quantitative Real-Time RT-PCR 

Estimation of the expression level of miR-21 and -155 was done by Quantitative real-time RT-PCR. miRNAs were extracted from tissue samples using a mirvana kit (Thermo Scientific, Waltham, MA, USA). Nanodrop^®^ spectrophotometer was used to measure the absorbance of the isolated miRNAs at 260 nm. Amplification and quantification of mature miRNAs were performed using Reverse Transcription–Polymerase Chain Reaction (RT-PCR) with TaqMan^®^ MicroRNA Assays, which planned to detect and accurately calculate mature microRNAs (micRNAs) using Applied Biosystems real-time PCR instruments. Throughout the target amplification step, the AmpliTaq^®^ Gold DNA polymerase amplifies target cDNA synthesized from the miRNA sample, using sequence-specific primers. The results were analyzed by relative quantification (Rq); it describes the change in the expression of the target gene relative to the reference group using the 2^−∆∆ct^ Method.

#### 2.4.7. Histopathological Examinations 

The kidneys from rats of different groups were assembled for histopathological examinations. These tissue samples were embedded in 10% neutral buffered formalin for 24 h. Afterwards, the samples were flushed with tap water. Afterwards, they were dehydrated, fixed in paraffin wax, and cut into 5 mm sections. Finally, they were stained with hematoxylin and eosin according to Gamble [49]. The tissues were scrutinized under a light microscope. 

### 2.5. Statistical Analysis 

Differences between groups were estimated using a one-way analysis of variance (ANOVA) and Tukey’s test was done for multiple comparisons. The probability of <0.05 was employed as the criterion for significance. The data were demonstrated as mean ± standard deviation (SD). The GraphPad Prism (ISI^®^, San Diego, CA, USA) software (version 9) was utilized to carry out all statistical analysis and graph illustrations. 

## 3. Results

### 3.1. Measurement of Relative Kidney Weight

In the current study, the administration of GNT significantly boosted the relative kidney weight by 30.34% with respect to the control group. Nevertheless, the administration of DIOS significantly diminished it by 15.5% of the GNT-treated rats’ values (Table 1).

### 3.2. Estimation of Renal Function Markers

Table 2 showed that GNT markedly elevated the BUN, SCr, and KIM-1 serum levels to 369.66, 806, and 308.44%, respectively, in comparison with the control group. DIOS pretreatment significantly lessened their values by 48.9, 70.6, and 50.5%, respectively, with respect to GNT-treated rats.

### 3.3. Determination of Renal Oxidative Stress Markers 

It was revealed by Table 3 that GNT significantly (*p* < 0.05) instigated a 4.27-fold increase in the renal MDA level with a 0.39- and 0.36-fold drop in the renal TAC and SOD levels, respectively, from the control animals’ values. In contrast, DIOS pretreatment instigated a 0.45-fold drop in renal MDA levels with a 1.64- and 2.16-fold rise in the renal TAC and SOD levels, respectively, with respect to GNT-treated rats.

### 3.4. Assessment of Nrf2 and HO-1 Expression in the Kidney 

**Figure 1:**
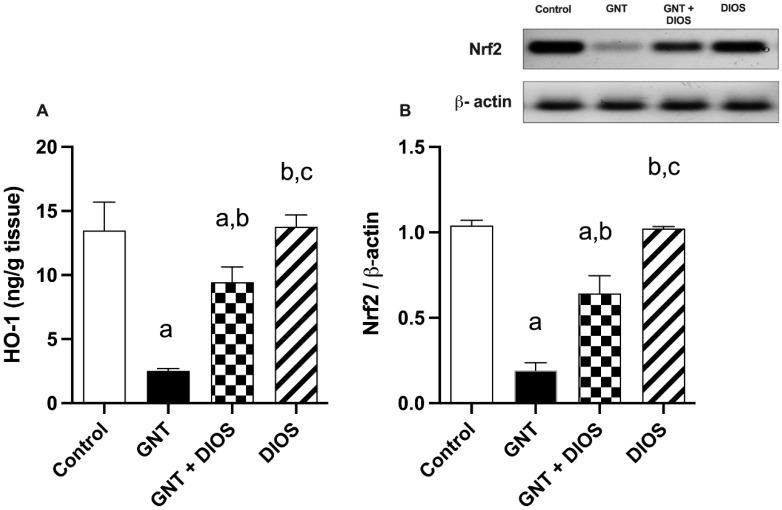
Impact of diosmin on the alterations in the kidney levels of HO-1 (**A**) and Nrf2 (**B**) induced by gentamicin: Values designates the mean ± SD (*n* = 6). a: statistically significant from the control group, b: statistically significant from the GNT group, c: statistically significant from the GNT + DIOS group, *p* < 0.05 determined with one-way ANOVA tailed by Tukey’s multiple comparisons test. Abbreviations: GNT, gentamicin; HO-1, heme oxygenase-1; Nrf2, nuclear factor E2-related factor 2; DIOS, diosmin.

By spotting Figure 1, GNT administration resulted in a substantial diminution in Nrf2 and HO-1 expression by 0.184- and 0.187-fold, respectively, with respect to the control group. However, DIOS pretreatment developed a significant increase by 3.36- and 3.745-fold in the Nrf2 and HO-1 expression, respectively, as compared with the GNT-treated rats.

### 3.5. Evaluation of Inflammatory Markers, p38-MAPK and NF-κB p65 Levels 

**Figure 2:**
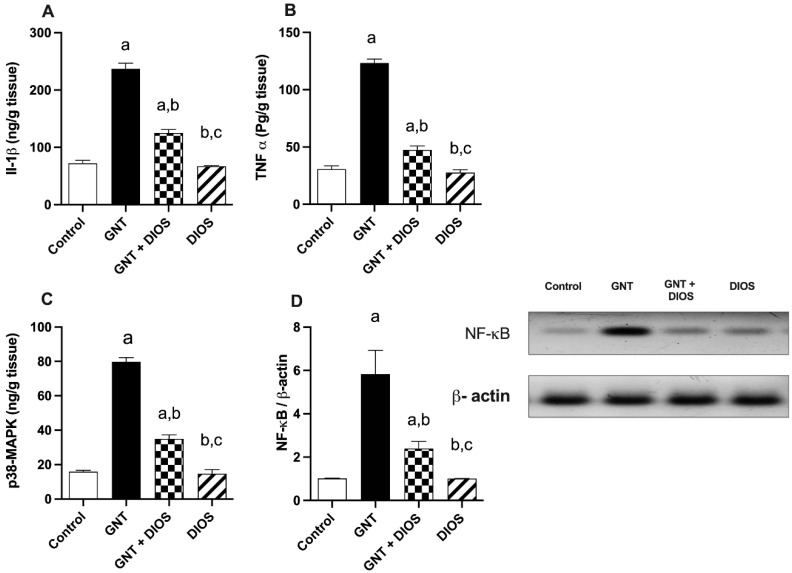
Impact of diosmin on renal inflammation induced by gentamicin (IL-1β (**A**), TNF-α (**B**), p38 MAPK (**C**) and NF-κB (**D**): the values designate the mean ± SD (*n* = 6). a: statistically significant from the control group, b: statistically significant from the GNT group, c: statistically significant from the GNT + DIOS group, *p* < 0.05 determined with one-way ANOVA tailed by Tukey’s multiple comparisons test. Abbreviations: GNT, gentamicin; IL-1β, interlukin-1 beta; NF-κB, nuclear factor-κappa B; p38 MAPK, p38 mitogen-activated protein kinase; DIOS, diosmin; TNF-α, tumor necrosis factor-alpha.

Figure 2 revealed that GNT resulted in a pointedly rise in the level of IL-1β and TNF-α, as well as the partaker proteins; p38-MAPK and NF-κB p65 to 401.6, 328.4, 503.3 and 573.5% respectively, in comparison with the control rats. Instead, DIOS administration noticeably diminished their values to 38.4, 52.8, 43.9 and 41.145% respectively as compared with the GNT-treated rats.

### 3.6. Estimation of Apoptotic Markers 

**Figure 3:**
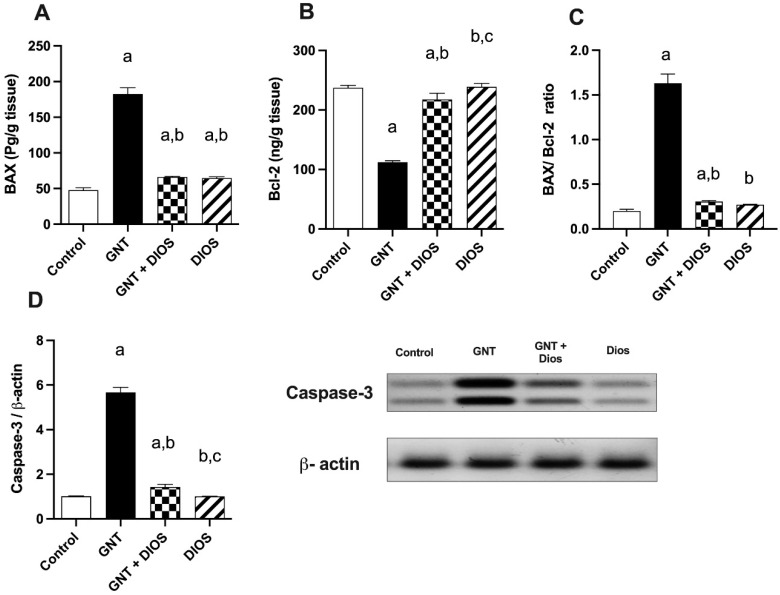
Impact of diosmin on the alterations in the renal apoptotic markers induced by gentamicin (BAX (**A**), Bcl-2 (**B**), BAX/ Bcl-2 ratio (**C**) and caspase-3 (**D**): The values designate the mean ± SD (*n* = 6). a: statistically significant from the control group, b: statistically significant from the GNT group, c: statistically significant from the GNT + DIOS group, *p* < 0.05 determined with one-way ANOVA tailed by Tukey’s multiple comparisons test. Abbreviations: BAX, Bcl-2-associated X protein; Bcl-2, B-cell lymphoma 2; GNT, gentamicin; DIOS, diosmin.

As shown in Figure 3, administration of GNT induced a considerable upsurge in the renal tissue level of BAX and Caspase-3 by 282.2 and 457.23%, respectively, and a marked-down surge in Bcl-2 level by 52.84% with respect to the control group. However, DIOS pretreatment markedly dropped the BAX and Caspase-3 levels by 63.7 and 74.75%, respectively, and markedly raised the Bcl2 level by 94.2% with respect to the GNT-treated group.

Undeniably, the BAX/Bcl-2 ratio is a crucial marker for the susceptibility of the cells to apoptosis. The current study unveiled a noteworthy rise in BAX/Bcl-2 ratio in GN-treated rats by 710.23% with respect to the control values. Nevertheless, when compared with GNT-treated rats, this ratio was markedly dropped via DIOS treatment by 81.29%.

### 3.7. Effect of Diosmin on microRNAs Expression

**Figure 4:**
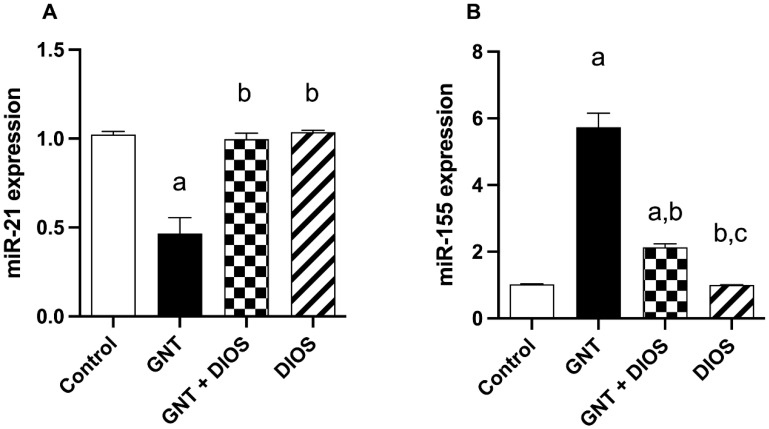
Impact of diosmin on the alterations in the renal expression of MiR-21 (**A**) and -155 (**B**) induced by gentamicin: The values designate the mean ± SD (*n* = 6). a: statistically significant from the control group, b: statistically significant from the GNT group, c: statistically significant from the GNT + DIOS group, *p* < 0.05 determined with one-way ANOVA tailed by Tukey’s multiple comparisons test. Abbreviations: GNT, gentamicin; MiR, micro-RNA; DIOS, diosmin.

From the data elucidated in Figure 4, GNT significantly induced a 0.46-fold decrease in miR-21 and a 5.62-fold upsurge in miR-155 expression from the control rats’ values. Pretreatment with DIOS significantly caused a 2.14-fold rise in miR-21 and a 0.37-fold drop in miR-155 expression with respect to the GNT-treated rats.

### 3.8. Effect of Diosmin Supplementation on Kidney Histology

**Figure 5:**
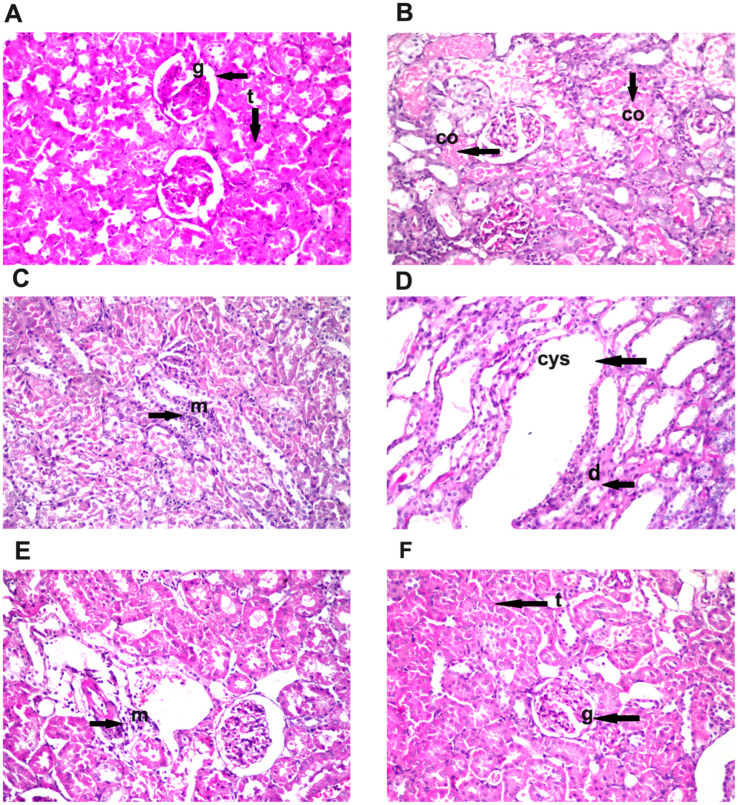
Descriptive photomicrographs of renal sections stained by hematoxylin-eosin (H and E) stain (magnification × 40) (*n* = 6). (**A**,**F**) Control and DIOS groups revealing normal histological appearance of the glomeruli (g) and tubules (t) at the cortex. (**B**) GNT group exhibiting coagulative necrosis (co) that was observed in a diffuse manner all over the lining tubular epithelium at the cortex. (**C**) GNT group presenting inflammatory cells infiltration (m) amongst the degenerated tubules at the cortex. (**D**) GNT group indicating cystic dilatation (cys) and degeneration (d) in the tubules at the corticomedullary portion. (**E**) GNT + DIOS group showing focal few inflammatory cells (m) infiltration amongst the tubules at the cortex.

Histopathological investigation of kidney sections from all treated groups was elucidated in Figure 5. Normal histological structure for glomeruli surrounded by numerous renal tubules was shown in the control and the DIOS-treated groups (Figure 5A,F). GNT treatment resulted in coagulative necrosis in a diffused manner all over the lining tubular epithelium at the cortex (Figure 5B) associated with inflammatory cells infiltration in between the degenerated tubules (Figure 5C) as well as cystic dilatation and degeneration in the tubules at the corticomedullary portion (Figure 5D). Pretreatment with DIOS (GNT + DIOS group) resulted in focal few inflammatory cells infiltration amongst the tubules at the cortex (Figure 5E). The semiquantitative histological score of the kidney sections is shown in Table 4.

## 4. Discussion

The kidneys are considered the prime target for chemical-induced toxicity due to their role in the excretion of toxicants and their metabolites [11]. Many therapeutic agents have been reported to induce nephrotoxicity in clinical use [50]. GNT, a commonly used aminoglycoside antibiotic, is one of the leading causes of drug-induced nephrotoxicity [51]. In this context, the current work was held to scrutinize the possible nephron-protective effect of DIOS in GNT-induced kidney injury. In the present investigation, rats administered with GNT displayed noticeable nephrotoxicity, revealed via the rise in the relative kidney weight, level of serum urea, creatinine, and KIM-1. Additionally, severe renal histological alterations demonstrated by coagulative necrosis of tubular epithelium, degenerated tubules, and inflammation were elucidated. These results reflect the nephrotoxic impact of gentamicin on the kidneys. Previous reports have shown similar results [4,15,52,53]. On the other hand, DIOS exhibited significant enhancement in renal functions reinforced by the ameliorations seen in the histological examination. Similar findings were observed in studies done by Rehman et al. [28] and El-Fawal et al. [34]. 

Several studies confirmed that GNT elevated the oxidative stress biomarkers and reduced the antioxidant biomarkers, supporting the hypothesis that oxidative stress contributes to GNT-induced renal toxicity [15,54,55]. Furthermore, HO-1 is an essential partaker protein of the body’s defense against oxidation. Nrf2 suppresses the oxidative injury response by regulating the antioxidant partaker’s protein expression, including the HO-1 gene [56]. Former studies found that Nrf2/HO-1 expression was down-regulated following GNT injection [9,10]. The current research unveiled that GNT caused drastic disruption of the renal oxidant/antioxidant status evidenced by a marked elevation in MDA level and reduction in both TAC and SOD levels as well as Nrf2 and HO-1 expression. Interestingly, co-treatment of GNT with DIOS restored the oxidant/antioxidant balance by decreasing the MDA level and increasing the TAC and SOD levels, along with Nrf2 and HO-1 expression. These outcomes are in line with earlier studies reporting the potent antioxidant effect of DIOS against different animal models [29,30,39]. Thus, these findings show that DIOS protected against GNT-induced renal oxidative injury by triggering the expression of Nrf2/HO-1. 

Results of the present work revealed that GNT-treated rats exhibited a significant elevation in the renal level of TNF-α and IL-1β, reflecting inflammatory progression. This finding is in good agreement with Cao et al. [57]. The DIOS anti-inflammatory effect was shown by ameliorating the increase in both TNF-α and IL-1β levels, which is in parallel with former studies [37,38].

Apoptosis plays a key role in numerous renal diseases and drug-induced nephrotoxicity [58]. In the current investigation, GNT caused a dramatic up-regulation in both caspase-3 and the pro-apoptotic BAX expression along with notable down-regulation in the anti-apoptotic Bcl-2 expression, suggesting that GNT-induced renal injury is associated with apoptotic pathway activation. Similar results were also detected in previous studies done by El Gamal et al. [59] and Sahu et al. [60]. On the other hand, DIOS provided reno-protection by attenuating the caspase-3 and BAX overexpression together with enhancing Bcl-2 expression. These outcomes are in accordance with different experimental studies demonstrating DIOS’ antiapoptotic effect [28,61,62].

To scrutinize further molecular mechanisms underlying the anti-inflammatory and anti-apoptotic responses brought by DIOS, renal expression of p38-MAPK and NF-κB were evaluated. Being involved in the inflammatory response and apoptosis, p38-MAPK is essential for the production of the pro-inflammatory cytokines, comprising IL-1β and TNF-α; it regulates NF-κB activation and controls cell apoptosis in renal injury [13]. NF-κB regulates various cellular responses such as innate immunity, inflammation, apoptosis, growth, and development [63]. It is expressed in renal impairment and involved in inflammatory responses [64,65]. Moreover, NF-κB activation in response to ROS could be implicated in GNT nephrotoxicity [66]. The current study showed that GNT boosted renal expression of p38-MAPK and NF-κB. These findings are matching with the results of former studies [14,67]. Nevertheless, pretreatment with DIOS markedly reversed these alterations, unveiling that the anti-inflammatory and anti-apoptotic effects of DIOS perceived in this study may be related to the downregulation of p38-MAPK and NF-κB. Our findings are in line with earlier research [29,30].

Dysfunctional miRNA expression has been implicated in the progression of kidney injury [68,69]. miR-21 is known to regulate apoptosis as well as inflammatory and fibrotic pathways in acute renal injury [70]. It was found to up-regulate the survival factor BCL2 and suppress the pro-apoptotic programmed cell death protein 4 (PDCD4) gene expression as well as the level of active caspase-3 in the kidneys of mice [71]. Similarly, various studies demonstrated the protective effect of miR-21 against renal ischemia-reperfusion injury by hindering the inflammatory response and apoptosis [72,73,74]. On the contrary, preceding in vivo and in vitro studies verified that miR-21 overexpression resulted in the up-regulation of pro-inflammatory cytokines, reflecting the dual role miR-21 could play in response to different stimuli [75,76]. Various reports have documented the role of miR-155 in the control of inflammatory reactions [23]. In the kidneys, elevated expression of miR-155 has been found in renal cancer, end-stage renal disease, immunological events, as well as in response to injury [22,77,78,79,80]. To further characterize the potential mechanistic pathway behind the reno-protective effect of DIOS against GNT-induced-renal damage, we assayed miR-21 and -155 expression with the qRT-PCR technique. In the current work, GNT-treated rats exhibited a marked decline in miR-21 expression together with a significant increase in miR-155 expression, which is in parallel with Saikumar et al. [23] and Mohamed et al. [81]. Interestingly, DIOS reversed the alterations in miR-21 and miR-155 gene expression induced by GNT indicating potential inhibition of apoptosis and inflammation. Far as we could tell, our study represents an unprecedented investigation of the role of DIOS in miR-21 and -155 expression in kidney tissue.

Taken together, our results demonstrated a reno-protective ability of DIOS versus GNT-induced renal damage, which may be ascribed to mitigation of the oxidative stress and amelioration of p38-MAPK and NF-κB pathways as well as miR-21 and -155 renal expression that subsequently engages in the inhibition of apoptosis and inflammation. These findings suggest that DIOS could serve as an adjuvant therapy with GNT to halt its nephrotoxic adverse effect.

## Figures and Tables

**Table 1:** Effect of DIOS on GNT-induced changes in relative kidney weight.

Groups	Relative Kidney Weight %
Control	0.369 ± 0.026
GNT	0.481 ± 0.085 ^a^
GNT + DIOS	0.406 ± 0.021 ^b^
DIOS	0.322 ± 0.034 ^b,c^

Values designate the mean ± SD (*n* = 6). a: statistically significant from the control group, b: statistically significant from the GNT group, c: statistically significant from the GNT + DIOS group, *p* < 0.05 determined with one-way ANOVA tailed by Tukey’s multiple comparisons test. Abbreviations: GNT, gentamicin; DIOS, diosmin.

**Table 2:** Effect of DIOS on GNT-induced alterations in renal function biomarkers.

Groups	BUN	SCr	KIM-1
Control	32.57 ± 2.421	0.175 ± 0.034	107.80 ± 3.794
GNT	120.40 ± 5.328 ^a^	1.417 ± 0.339 ^a^	332.50 ± 28.120 ^a^
GNT + DIOS	61.52 ± 7.413 ^a,b^	0.416 ± 0.018 ^b^	164.50 ± 5.526 ^a,b^
DIOS	40.67 ± 5.901 ^b,c^	0.194 ± 0.020 ^b^	104.50 ± 6.237 ^b,c^

The values designate the mean ± SD (*n* = 6). a: statistically significant from the control group, b: statistically significant from the GNT group, c: statistically significant from the GNT + DIOS group, *p* < 0.05 determined with one-way ANOVA tailed by Tukey’s multiple comparisons test. Abbreviations: BUN, blood urea nitrogen; GNT, gentamicin; KIM-1, kidney injury molecule-1; DIOS, diosmin; SCr, serum creatinine.

**Table 3:** Effect of DIOS on GNT-induced renal oxidative stress.

Groups	MDA	TAC	SOD
Control	44.50 ± 4.472	79.58 ± 8.695	42.68 ± 4.377
GNT	190.2 ± 13.040 ^a^	31.12 ± 4.481 ^a^	15.51 ± 1.350 ^a^
GNT + DIOS	84.88 ± 9.566 ^a,b^	51.20 ± 3.913 ^a,b^	33.45 ± 2.931 ^a,b^
DIOS	50.91 ± 9.671 ^b,c^	70.82 ± 2.971 ^b,c^	44.37 ± 3.258 ^b,c^

The values designate the mean ± SD (*n* = 6). a: statistically significant from the control group, b: statistically significant from the GNT group, c: statistically significant from the GNT + DIOS group, *p* < 0.05 determined with one-way ANOVA tailed by Tukey’s multiple comparisons test. Abbreviations: GNT, gentamicin; MDA, malondialdehyde; DIOS, diosmin; SOD, superoxide dismutase; TAC, total antioxidant capacity.

**Table 4:** Semiquantitative histological score of the kidney sections.

Groups	Tubular Coagulative Necrosis	Perivascular Edema and Inflammatory Cells Infiltration	Tubular Degeneration	Focal Inflammatory Cells Infiltration
Control	-	-	-	-
GNT	+++	+	++	++
GNT+ DIOS	-	-	-	+
DIOS	-	-	-	-

At least, six fields for each kidney section were examined and assigned for the severity of changes using a score of (-) for negative, (+) for mild, (++) for moderate or (+++) for severe histopathological alterations (*n* = 6). Abbreviations: GNT, gentamicin; DIOS, diosmin.

## Data Availability

Data are contained within the article.

## References

[1] Balakumar P., Rohilla A., Thangathirupathi A. (2010). Gentamicin-Induced Nephrotoxicity: Do We Have a Promising Therapeutic Approach to Blunt It?. Pharmacol. Res..

[2] Stojiljkovic N., Veljkovic S., Mihailovic D., Stoiljkovic M., Radenkovic M., Rankovic G., Randjelovic P. (2009). Protective Effects of Pentoxifylline Treatment on Gentamicin-Induced Nephrotoxicity in Rats. Ren. Fail..

[3] Medić B., Stojanović M., Rovčanin B., Kekić D., Škodrić S.R., Jovanović G.B., Vujović K.S., Divac N., Stojanović R., Radenković M. (2019). Pioglitazone Attenuates Kidney Injury in an Experimental Model of Gentamicin-Induced Nephrotoxicity in Rats. Sci. Rep..

[4] Udupa V., Prakash V. (2019). Gentamicin Induced Acute Renal Damage and Its Evaluation Using Urinary Biomarkers in Rats. Toxicol. Rep..

[5] Juan S.H., Chen C.H., Hsu Y.H., Hou C.C., Chen T.H., Lin H., Chu Y.L., Sue Y.M. (2007). Tetramethylpyrazine Protects Rat Renal Tubular Cell Apoptosis Induced by Gentamicin. Nephrol. Dial. Transplant..

[6] Lee I.C., Kim S.H., Lee S.M., Baek H.S., Moon C., Kim S.H., Park S.C., Kim H.C., Kim J.C. (2012). Melatonin Attenuates Gentamicin-Induced Nephrotoxicity and Oxidative Stress in Rats. Arch. Toxicol..

[7] Jaikumkao K., Pongchaidecha A., Thongnak L.O., Wanchai K., Arjinajarn P., Chatsudthipong V., Chattipakorn N., Lungkaphin A. (2016). Amelioration of Renal Inflammation, Endoplasmic Reticulum Stress and Apoptosis Underlies the Protective Effect of Low Dosage of Atorvastatin in Gentamicin-Induced Nephrotoxicity. PLoS ONE.

[8] Yu H., Chen B., Ren Q. (2019). Baicalin Relieves Hypoxia-Aroused H9c2 Cell Apoptosis by Activating Nrf2/HO-1-Mediated HIF1α/BNIP3 Pathway. Artif. Cells Nanomed. Biotechnol..

[9] He L., Peng X., Zhu J., Liu G., Chen X., Tang C., Liu H., Liu F., Peng Y. (2015). Protective Effects of Curcumin on Acute Gentamicin-Induced Nephrotoxicity in Rats. Can. J. Physiol. Pharmacol..

[10] Subramanian P., Anandan R., Jayapalan J.J., Hashim O.H. (2015). Hesperidin Protects Gentamicin-Induced Nephrotoxicity via Nrf2/HO-1 Signaling and Inhibits Inflammation Mediated by NF-ΚB in Rats. J. Funct. Foods.

[11] Nassan M.A., Soliman M.M., Aldhahrani A., Althobaiti F., Alkhedaide A.Q. (2021). Ameliorative Impacts of Glycyrrhiza Glabra Root Extract against Nephrotoxicity Induced by Gentamicin in Mice. Food Sci. Nutr..

[12] Volpini R.A., Balbi A.P.C., Costa R.S., Coimbra T.M. (2006). Increased Expression of P38 Mitogen-Activated Protein Kinase Is Related to the Acute Renal Lesions Induced by Gentamicin. Braz. J. Med. Biol. Res..

[13] Ozbek E., Cekmen M., Ilbey Y.O., Simsek A., Polat E.C., Somay A. (2009). Atorvastatin Prevents Gentamicin-Induced Renal Damage in Rats through the Inhibition of P38-MAPK and NF-KB Pathways. Ren. Fail..

[14] Wu D., Luo N., Wang L., Zhao Z., Bu H., Xu G., Yan Y., Che X., Jiao Z., Zhao T. (2017). Hydrogen Sulfide Ameliorates Chronic Renal Failure in Rats by Inhibiting Apoptosis and Inflammation through ROS/MAPK and NF-ΚB Signaling Pathways. Sci. Rep..

[15] Ahmed H.I., Mohamed E.A. (2019). Candesartan and Epigallocatechin-3-Gallate Ameliorate Gentamicin-Induced Renal Damage in Rats through P38-MAPK and NF-ΚB Pathways. J. Biochem. Mol. Toxicol..

[16] O’Brien J., Hayder H., Zayed Y., Peng C. (2018). Overview of MicroRNA Biogenesis, Mechanisms of Actions, and Circulation. Front. Endocrinol..

[17] Jones T.F., Bekele S., O’Dwyer M.J., Prowle J.R. (2018). MicroRNAs in Acute Kidney Injury. Nephron.

[18] Paul P., Chakraborty A., Sarkar D., Langthasa M., Rahman M., Bari M., Singha R.K.S., Malakar A.K., Chakraborty S. (2018). Interplay between MiRNAs and Human Diseases. J. Cell. Physiol..

[19] Huang W. (2017). MicroRNAs: Biomarkers, Diagnostics, and Therapeutics. Methods Mol. Biol..

[20] Suo A., Lan Z., Lu C., Zhao Z., Pu D., Wu X., Jiang B., Zhou N., Ding H., Zhou D. (2021). Characterizing MicroRNAs and Their Targets in Different Organs of Camellia Sinensis Var. Assamica. Genomics.

[21] Wei Q., Mi Q.S., Dong Z. (2013). The Regulation and Function of Micrornas in Kidney Diseases. IUBMB Life.

[22] Wang G., Kwan B.C.H., Lai F.M.M., Chow K.M., Li P.K.T., Szeto C.C. (2011). Elevated Levels of MiR-146a and MiR-155 in Kidney Biopsy and Urine from Patients with IgA Nephropathy. Dis. Markers.

[23] Saikumar J., Hoffmann D., Kim T.M., Gonzalez V.R., Zhang Q., Goering P.L., Brown R.P., Bijol V., Park P.J., Waikar S.S. (2012). Expression, Circulation, and Excretion Profile of MicroRNA-21, -155, and -18a Following Acute Kidney Injury. Toxicol. Sci..

[24] Glowacki F., Savary G., Gnemmi V., Buob D., van der Hauwaert C., Lo-Guidice J.M., Bouyé S., Hazzan M., Pottier N., Perrais M. (2013). Increased Circulating MiR-21 Levels Are Associated with Kidney Fibrosis. PLoS ONE.

[25] Zhou H., Hasni S.A., Perez P., Tandon M., Jang S.I., Zheng C., Kopp J.B., Austin H., Balow J.E., Alevizos I. (2013). MiR-150 Promotes Renal Fibrosis in Lupus Nephritis by Downregulating SOCS1. J. Am. Soc. Nephrol..

[26] Sun Y., Peng R., Peng H., Liu H., Wen L., Wu T., Yi H., Li A., Zhang Z. (2016). MiR-451 Suppresses the NF-KappaB-Mediated Proinflammatory Molecules Expression through Inhibiting LMP7 in Diabetic Nephropathy. Mol. Cell. Endocrinol..

[27] Zheng Y., Zhang R., Shi W., Li L., Liu H., Chen Z., Wu L. (2020). Metabolism and Pharmacological Activities of the Natural Health-Benefiting Compound Diosmin. Food Funct..

[28] Rehman M.U., Tahir M., Quaiyoom Khan A., Khan R., Lateef A., Hamiza O.O., Ali F., Sultana S. (2013). Diosmin Protects against Trichloroethylene-Induced Renal Injury in Wistar Rats: Plausible Role of P53, Bax and Caspases. Br. J. Nutr..

[29] Ali F.E.M., Bakr A.G., Abo-youssef A.M., Azouz A.A., Hemeida R.A.M. (2018). Targeting Keap-1/Nrf-2 Pathway and Cytoglobin as a Potential Protective Mechanism of Diosmin and Pentoxifylline against Cholestatic Liver Cirrhosis. Life Sci..

[30] Ali F.E.M., Azouz A.A., Bakr A.G., Abo-youssef A.M., Hemeida R.A.M. (2018). Hepatoprotective Effects of Diosmin and/or Sildenafil against Cholestatic Liver Cirrhosis: The Role of Keap-1/Nrf-2 and P38-MAPK/NF-ΚB/INOS Signaling Pathway. Food Chem. Toxicol..

[31] Hsu C.C., Lin M.H., Cheng J.T., Wu M.C. (2017). Antihyperglycaemic Action of Diosmin, a Citrus Flavonoid, Is Induced through Endogenous β-Endorphin in Type I-like Diabetic Rats. Clin. Exp. Pharmacol. Physiol..

[32] Lewinska A., Adamczyk-Grochala J., Kwasniewicz E., Deregowska A., Wnuk M. (2017). Diosmin-Induced Senescence, Apoptosis and Autophagy in Breast Cancer Cells of Different P53 Status and ERK Activity. Toxicol. Lett..

[33] Carballo-Villalobos A.I., González-Trujano M.E., Pellicer F., Alvarado-Vásquez N., López-Muñoz F.J. (2018). Central and Peripheral Anti-Hyperalgesic Effects of Diosmin in a Neuropathic Pain Model in Rats. Biomed. Pharmacother..

[34] El-Fawal R., el Fayoumi H.M., Mahmoud M.F. (2018). Diosmin and Crocin Alleviate Nephropathy in Metabolic Syndrome Rat Model: Effect on Oxidative Stress and Low Grade Inflammation. Biomed. Pharmacother..

[35] Nirumand M.C., Hajialyani M., Rahimi R., Farzaei M.H., Zingue S., Nabavi S.M., Bishayee A. (2018). Dietary Plants for the Prevention and Management of Kidney Stones: Preclinical and Clinical Evidence and Molecular Mechanisms. Int. J. Mol. Sci..

[36] Schlottfeldt F.D.S., Fernandes S.M., Martins D.M., Cordeiro P., da Fonseca C.D., Watanabe M., Vattimo M.D.F.F. (2015). Prevention of Amphotericin B Nephrotoxicity through Use of Phytotherapeutic Medication. Rev. Da Esc. De Enferm..

[37] Ahmed S., Mundhe N., Borgohain M., Chowdhury L., Kwatra M., Bolshette N., Ahmed A., Lahkar M. (2016). Diosmin Modulates the NF-KB Signal Transduction Pathways and Downregulation of Various Oxidative Stress Markers in Alloxan-Induced Diabetic Nephropathy. Inflammation.

[38] Abdel-Daim M.M., Khalifa H.A., Abushouk A.I., Dkhil M.A., Al-Quraishy S.A. (2017). Diosmin Attenuates Methotrexate-Induced Hepatic, Renal, and Cardiac Injury: A Biochemical and Histopathological Study in Mice. Oxid. Med. Cell. Longev..

[39] Ali F.E.M., Sayed A.M., El-Bahrawy A.H., Omar Z.M.M., Hassanein E.H.M. (2021). Targeting KEAP1/Nrf2, AKT, and PPAR-γ Signals as a Potential Protective Mechanism of Diosmin against Gentamicin-Induced Nephrotoxicity. Life Sci..

[40] Ali N., AlAsmari A.F., Imam F., Ahmed M.Z., Alqahtani F., Alharbi M., AlSwayyed M., AlAsmari F., Alasmari M., Alshammari A. (2021). Protective effect of diosmin against doxorubicin-induced nephrotoxicity. Saudi J. Biol. Sci..

[41] El-Agroudy N.N., El-Naga R.N., El-Razeq R.A., El-Demerdash E. (2016). Forskolin, a Hedgehog Signalling Inhibitor, Attenuates Carbon Tetrachloride-Induced Liver Fibrosis in Rats. Br. J. Pharmacol..

[42] Darwish M.A., Abo-Youssef A.M., Khalaf M.M., Abo-Saif A.A., Saleh I.G., Abdelghany T.M. (2017). Vitamin E Mitigates Cisplatin-Induced Nephrotoxicity Due to Reversal of Oxidative/Nitrosative Stress, Suppression of Inflammation and Reduction of Total Renal Platinum Accumulation. J. Biochem. Mol. Toxicol..

[43] Jung D., Biggs H., Erikson J., Ledyard P.U. (1975). New Colorimetric Reaction for End Point, Continuous Flow, and Kinetic Measurement of Urea. Clin. Chem..

[44] Wang J.J., Zhang S.X., Mott R., Knapp R.R., Cao W., Lau K., Ma J.X. (2006). Salutary Effect of Pigment Epithelium-Derived Factor in Diabetic Nephropathy: Evidence for Antifibrogenic Activities. Diabetes.

[45] Ohkawa H., Ohishi N., Yagi K. (1979). Assay for Lipid Peroxides in Animal Tissues by Thiobarbituric Acid Reaction. Anal. Biochem..

[46] Nishikimi M., Roa N.A., Yogi K. (1972). Determination of Superoxide Dismutase in Tissue Homogenate. Biochem. Bioph. Res. Common..

[47] Koracevic D., Koracevic G., Djordjevic V., Andrejevic S., Cosic V. (2001). Method for the Measurement of Antioxidant Activity in Human Fluids. J. Clin. Pathol..

[48] Bradford M. (1976). A Rapid and Sensitive Method for the Quantitation of Microgram Quantities of Protein Utilizing the Principle of Protein-Dye Binding. Anal. Biochem..

[49] Gamble M., Bancroft J., Gamble M. (2008). The Hematoxylins and Eosin. Theory and Practice of Histological Techniques.

[50] Perazella M.A. (2018). Pharmacology behind Common Drug Nephrotoxicities. Clin. J. Am. Soc. Nephrol..

[51] Randjelović P., Veljković S., Stojiljković N., Sokolović D., Ilić I. (2017). Gentamicin Nephrotoxicity in Animals: Current Knowledge and Future Perspectives. EXCLI J..

[52] Sabbisetti V.S., Waikar S.S., Antoine D.J., Smiles A., Wang C., Ravisankar A., Ito K., Sharma S., Ramadesikan S., Lee M. (2014). Blood Kidney Injury Molecule-1 Is a Biomarker of Acute and Chronic Kidney Injury and Predicts Progression to ESRD in Type I Diabetes. J. Am. Soc. Nephrol..

[53] Al-Kuraishy H.M., Al-Gareeb A.I., Al-Naimi M.S. (2019). Renoprotective Effect of Irbesartan in a Rat Model of Gentamicin-Induced Nephrotoxicity: Role of Oxidative Stress. J. Lab. Physicians.

[54] Igwebuike C., Yaglom J., Huiting L., Feng H., Campbell J.D., Wang Z., Havasi A., Pimentel D., Sherman M.Y., Borkan S.C. (2020). Cross Organelle Stress Response Disruption Promotes Gentamicin-Induced Proteotoxicity. Cell Death Dis..

[55] Bulboacă A.E., Porfire A., Bolboacă S.D., Nicula C.A., Feștilă D.G., Roman A., Râjnoveanu R.M., Râjnoveanu A., Dogaru G., Boarescu P.M. (2021). Protective Effects of Liposomal Curcumin on Oxidative Stress/Antioxidant Imbalance, Metalloproteinases 2 and -9, Histological Changes and Renal Function in Experimental Nephrotoxicity Induced by Gentamicin. Antioxidants.

[56] Deng J.S., Jiang W.P., Chen C.C., Lee L.Y., Li P.Y., Huang W.C., Liao J.C., Chen H.Y., Huang S.S., Huang G.J. (2020). Cordyceps Cicadae Mycelia Ameliorate Cisplatin-Induced Acute Kidney Injury by Suppressing the TLR4/NF-κB/MAPK and Activating the HO-1/Nrf2 and Sirt-1/AMPK Pathways in Mice. Oxid. Med. Cell. Longev..

[57] Cao L., Zhi D., Han J., Kumar Sah S., Xie Y. (2019). Combinational Effect of Curcumin and Metformin against Gentamicin-Induced Nephrotoxicity: Involvement of Antioxidative, Anti-Inflammatory and Antiapoptotic Pathway. J. Food Biochem..

[58] Servais H., Ortiz A., Devuyst O., Denamur S., Tulkens P.M., Mingeot-Leclercq M.P. (2008). Renal Cell Apoptosis Induced by Nephrotoxic Drugs: Cellular and Molecular Mechanisms and Potential Approaches to Modulation. Apoptosis.

[59] El Gamal A.A., Alsaid M.S., Raish M., Al-Sohaibani M., Al-Massarani S.M., Ahmad A., Hefnawy M., Al-Yahya M., Basoudan O.A., Rafatullah S. (2014). Beetroot (Beta Vulgaris L.) Extract Ameliorates Gentamicin-Induced Nephrotoxicity Associated Oxidative Stress, Inflammation, and Apoptosis in Rodent Model. Mediat. Inflamm..

[60] Sahu B.D., Tatireddy S., Koneru M., Borkar R.M., Kumar J.M., Kuncha M., Srinivas R., Shyam Sunder R., Sistla R. (2014). Naringin Ameliorates Gentamicin-Induced Nephrotoxicity and Associated Mitochondrial Dysfunction, Apoptosis and Inflammation in Rats: Possible Mechanism of Nephroprotection. Toxicol. Appl. Pharmacol..

[61] Shalkami A.S., Hassan M.I.A., Bakr A.G. (2018). Anti-Inflammatory, Antioxidant and Anti-Apoptotic Activity of Diosmin in Acetic Acid-Induced Ulcerative Colitis. Hum. Exp. Toxicol..

[62] Mahgoub S., Sallam A.O., Sarhan H.K.A., Ammar A.A.A., Soror S.H. (2020). Role of Diosmin in protection against the oxidative stress induced damage by gamma-radiation in Wistar albino rats. Regul. Toxicol. Pharmacol..

[63] Kumar D., Singla S.K., Puri V., Puri S. (2015). The Restrained Expression of NF-KB in Renal Tissue Ameliorates Folic Acid Induced Acute Kidney Injury in Mice. PLoS ONE.

[64] Sherif I.O., Al-Mutabagani L.A., Alnakhli A.M., Sobh M.A., Mohammed H.E. (2015). Renoprotective Effects of Angiotensin Receptor Blocker and Stem Cells in Acute Kidney Injury: Involvement of Inflammatory and Apoptotic Markers. Exp. Biol. Med..

[65] Ansari M.A., Raish M., Ahmad A., Alkharfy K.M., Ahmad S.F., Attia S.M., Alsaad A.M.S., Bakheet S.A. (2017). Sinapic Acid Ameliorate Cadmium-Induced Nephrotoxicity: In Vivo Possible Involvement of Oxidative Stress, Apoptosis, and Inflammation via NF-ΚB Downregulation. Environ. Toxicol. Pharmacol..

[66] Hassanein E.H.M., Ali F.E.M., Kozman M.R., Abd El-Ghafar O.A.M. (2021). Umbelliferone Attenuates Gentamicin-Induced Renal Toxicity by Suppression of TLR-4/NF-ΚB-P65/NLRP-3 and JAK1/STAT-3 Signaling Pathways. Environ. Sci. Pollut. Res..

[67] Babaeenezhad E., Hadipour Moradi F., Rahimi Monfared S., Fattahi M.D., Nasri M., Amini A., Dezfoulian O., Ahmadvand H. (2021). D-Limonene Alleviates Acute Kidney Injury Following Gentamicin Administration in Rats: Role of NF- κ B Pathway, Mitochondrial Apoptosis, Oxidative Stress, and PCNA. Oxid. Med. Cell. Longev..

[68] Gutiérrez-Escolano A., Santacruz-Vázquez E., Gómez-Pérez F. (2015). Dysregulated MicroRNAs Involved in Contrast-Induced Acute Kidney Injury in Rat and Human. Ren. Fail..

[69] Liu X.J., Hong Q., Wang Z., Yu Y.Y., Zou X., Xu L.H. (2015). MicroRNA-34a Suppresses Autophagy in Tubular Epithelial Cells in Acute Kidney Injury. Am. J. Nephrol..

[70] Li Y.F., Jing Y., Hao J., Frankfort N.C., Zhou X., Shen B., Liu X., Wang L., Li R. (2013). MicroRNA-21 in the Pathogenesis of Acute Kidney Injury. Protein Cell.

[71] Hu H., Jiang W., Xi X., Zou C., Ye Z. (2014). MicroRNA-21 Attenuates Renal Ischemia Reperfusion Injury via Targeting Caspase Signaling in Mice. Am. J. Nephrol..

[72] Godwin J.G., Ge X., Stephan K., Jurisch A., Tullius S.G., Iacomini J. (2010). Identification of a MicroRNA Signature of Renal Ischemia Reperfusion Injury. Proc. Natl. Acad. Sci. USA.

[73] Xu X., Kriegel A.J., Liu Y., Usa K., Mladinov D., Liu H., Fang Y., Ding X., Liang M. (2012). Delayed Ischemic Preconditioning Contributes to Renal Protection by Upregulation of MiR-21. Kidney Int..

[74] Zhang W., Shu L. (2016). Upregulation of MiR-21 by Ghrelin Ameliorates Ischemia/Reperfusion-Induced Acute Kidney Injury by Inhibiting Inflammation and Cell Apoptosis. DNA Cell Biol..

[75] Sheedy F.J., Palsson-Mcdermott E., Hennessy E.J., Martin C., O’Leary J.J., Ruan Q., Johnson D.S., Chen Y., O’Neill L.A.J. (2010). Negative Regulation of TLR4 via Targeting of the Proinflammatory Tumor Suppressor PDCD4 by the MicroRNA MiR-21. Nat. Immunol..

[76] Ando Y., Yang G.X., Kenny T.P., Kawata K., Zhang W., Huang W., Leung P.S.C., Lian Z.X., Okazaki K., Ansari A.A. (2013). Overexpression of MicroRNA-21 Is Associated with Elevated pro-Inflammatory Cytokines in Dominant-Negative TGF-β Receptor Type II Mouse. J. Autoimmun..

[77] Wang H., Peng W., Shen X., Huang Y., Ouyang X., Dai Y. (2012). Circulating Levels of Inflammation-Associated Mir-155 and Endothelial-Enriched Mir-126 in Patients with End-Stage Renal Disease. Braz. J. Med. Biol. Res..

[78] Gao Y., Ma X., Yao Y., Li H., Fan Y., Zhang Y., Zhao C., Wang L., Ma M., Lei Z. (2016). MiR-155 Regulates the Proliferation and Invasion of Clear Cell Renal Cell Carcinoma Cells by Targeting E2F2. Oncotarget.

[79] Zununi Vahed S., Poursadegh Zonouzi A., Ghanbarian H., Ghojazadeh M., Samadi N., Omidi Y., Ardalan M. (2017). Differential Expression of Circulating MiR-21, MiR-142-3p and MiR-155 in Renal Transplant Recipients with Impaired Graft Function. Int. Urol. Nephrol..

[80] Chen S., Shan J., Niu W., Lin F., Liu S., Wu P., Sun L., Lu W., Jiang G. (2018). Micro RNA-155 Inhibitor as a Potential Therapeutic Strategy for the Treatment of Acute Kidney Injury (AKI): A Nanomedicine Perspective. RSC Adv..

[81] Mohamed D.I., Khairy E., Saad S.S.T., Habib E.K., Hamouda M.A. (2019). Potential Protective Effects of Dapagliflozin in Gentamicin Induced Nephrotoxicity Rat Model via Modulation of Apoptosis Associated MiRNAs. Gene.

